# Preschoolers’ Technology-Assessed Physical Activity and Cognitive Function: A Cross-Sectional Study

**DOI:** 10.3390/jcm7050108

**Published:** 2018-05-08

**Authors:** Minghui Quan, Hanbin Zhang, Jiayi Zhang, Tang Zhou, Jinming Zhang, Guanggao Zhao, Hui Fang, Shunli Sun, Ru Wang, Peijie Chen

**Affiliations:** 1 School of Kinesiology, Shanghai University of Sport, Shanghai 200438, China; quanminghui@163.com (M.Q.); zhb20092009@163.com (H.Z.); tzhou_1023@foxmail.com (T.Z.); ffanghui@163.com (H.F.); sunsl087@outlook.com (S.S.); 2 Editorial Department of Medicine and Health, China Science Publishing and Media Ltd., Shanghai 200032, China; jiayi_0827@126.com; 3 Department of Kinesiology, College of Sport Medicine and Rehabilitation, Taishan Medical University, Taian 271016, China; jmzhang@tsmc.edu.cn; 4 Department of Physical Education, Nanchang University, Nanchang 330031, China; zhaogg2002@163.com

**Keywords:** motor activity, intelligence quotient, young children

## Abstract

Early childhood is a critical period for development of cognitive function, but research on the association between physical activity and cognitive function in preschool children is limited and inconclusive. This study aimed to examine the association between technology-assessed physical activity and cognitive function in preschool children. A cross-sectional analysis of baseline data from the Physical Activity and Cognitive Development Study was conducted in Shanghai, China. Physical activity was measured with accelerometers for 7 consecutive days, and cognitive functions were assessed using the Chinese version of Wechsler Young Children Scale of Intelligence (C-WYCSI). Linear regression analyses were used to assess the association between physical activity and cognitive function. A total of 260 preschool children (boys, 144; girls, 116; mean age: 57.2 ± 5.4 months) were included in analyses for this study. After adjusting for confounding factors, we found that Verbal Intelligence Quotient, Performance Intelligence Quotient, and Full Intelligence Quotient were significantly correlated with light physical activity, not moderate to vigorous physical activity, in boys. Standardized coefficients were 0.211, 0.218, and 0.242 (all *p* < 0.05) in three different models, respectively. However, the correlation between physical activity and cognitive functions were not significant in girls (*p* > 0.05). These findings suggest that cognitive function is apparently associated with light physical activity in boys. Further studies are required to clarify the sex-specific effect on physical activity and cognitive functions.

## 1. Introduction

Cognitive function is the ability to process information acquired from individual surroundings by the brain and includes the ability to learn and remember information, organize, plan and problem-solve, focus, maintain and shift attention, and understand and use language [1]. The stage of childhood is a critical period to develop cognitive function, as cognitive function in childhood may be an important indicator for future physical health, mental health, wealth, and public safety [2]. Therefore, identifying related factors in the development of cognitive function in childhood has drawn researchers’ attention due to its substantial benefits extending into adulthood.

Physical activity (PA), a component of lifestyle, is considered to be a potentially important factor in altering our brain health and mental function [3]. In recent years, many studies have examined the effects of PA on cognitive function. There is an increasing body of evidence suggesting that in children and adolescents, PA, particularly moderate to vigorous physical activity (MVPA), is closely associated with cognitive functions [4,5]. Furthermore, a systematic review concluded that there is a positive association between PA and cognitive function in children [6]. Animal evidence suggests that aerobic exercise can enhance human brain structure, prevent age-related brain tissue loss, and improve cognitive performance [7], so increased PA can enhance brain function. Previous human studies also indicated that PA in schools may enhance academic attainment, and higher levels of physical fitness in children may be associated with improved neurocognitive processing [8]. Increased physical activity may therefore provide cognitive and educational benefits across childhood and adolescence. So, moderate and vigorous PA was recommended for children and adolescents, and the importance of establishing healthy and appropriate behaviors in children is crucial for long-term effects. However, different types, amounts, and frequencies of PA were adopted in these studies. Moreover, in preschool stage, a key period for personality development, to the best of our knowledge, studies assessing the association between PA and cognitive functions is limited. Furthermore, previous trials were individually underpowered or primarily of weak quality to address this issue, and the few observational studies addressing it have mixed results [9].

Therefore, in the current study, the aim is to examine the PA conditions in preschool children and the association between the technology-assessed PA and cognitive functions, while adjusting for confounding factors that associated with cognitive function on the basis of previous studies, such as children’s cardiorespiratory fitness, daily behavior, and mother’s education [6,10,11].

## 2. Methods

### 2.1. Participants

This cross-sectional study is a baseline data analysis from The Physical Activity and Cognitive Function Study (Trial Registration: ChiCTR-OOC-15007439) [12]. A total of 346 (boys, 201; girls, 145) preschool children were recruited from seven urban kindergartens in Shanghai, China. All the parents/guardians of potential participants have been fully informed of the protocol and aims of the study by parents’ meeting held in the kindergarten. Signed informed consent forms were obtained from parents/guardians of the participants before this study began. The protocol was also approved by The Ethics Advisory Committee of Shanghai University of Sport.

### 2.2. Measures and Procedures

Participants’ cognitive functions were evaluated using a short form of the Chinese version of Wechsler Young Children Scale of Intelligence (C-WYCSI) [13], due to constraints in assessment time. The short form consisted of four subtests, taking approximately 30 min to complete, and was also widely adopted in previous studies investigating cognitive function [14,15]. Furthermore, the short form of C-WYCSI was validated and the association of its scores and estimated Full Intelligence Quotient (FIQ) was also confirmed in our pilot study for preschool children (*n* = 31, *r* = 0.90, *p* < 0.01). The short-form items consisted of two tests: the Verbal Intelligence Quotient (VIQ: Information and Vocabulary) test and the Performance Intelligence Quotient (PIQ: Picture Completion and Block Design) test. The Information subtest involved asking participants to answer questions about everyday knowledge; participants received a 1 or 0 score for each correct or incorrect answer (total, 0–23 scores). For the Vocabulary subtest, children were asked to identify the true answer from four pictures corresponding to the word instructed by the tester; children scored 1 or 0 for each correct or incorrect response (total, 0–44 scores). The Picture Completion subtest required children to identify and point out the missing part of the picture; children received a 1 or 0 score for each correct or incorrect answer (total, 0–25 scores). Finally, the Block Design subtest included a design either from the tester or the test booklet; children scored from 0 to 4 depending on how quickly they completed each design (total, 0–29 scores). After assessment, raw scores were converted to standard scores based on the instruction manual. Standardized scores of VIQ and PIQ were equal to the sum of two Verbal and Performance subtest scores, respectively. FIQ was estimated using weighted scores of each subtest according to the instruction manual (normal mean = 100, SD = 15.0). Children were divided into five groups based on their FIQ scores: significantly below normal, slightly below normal, normal, slightly above normal, and significantly above normal were defined as <70, 70–<90, 90–110, >110–130, and >130 scores, respectively.

Physical activity was measured over 7 consecutive days during waking hours with ActiGraph accelerometers (GT3X^+^, ActiGraph, Pensacola, FL, USA) on the right hip. The activity was captured by a 1 s sampling interval and categorized as either light or moderate to vigorous PA (LPA, MVPA) based on cutoff counts developed by Pate and colleagues for preschool children [16]. LPA corresponded with 101–1679 counts per minute (CPMs) and MVPA was equal or greater than 1680 CPMs. Individual data were validated into analyses when participating in at least 3 days (including 1 weekend day) of monitored PA, with a minimum of 8 h each day [17].

Children characteristics included sex, ages (months), heights, body weights, mother’s education, family structures, and household income. Body mass index (BMI) was calculated using the formula weight/height^2^ (kg/m^2^) and BMI status was classified as normal, overweight, and obese using the cut points developed by International Obesity Task Force (IOTF) [18]. Mother’s education was considered as a critical influence on children’s cognitive function [11], and the education level was divided into six groups: less than high school, high school, some college/associate’s degree, bachelor degree, master’s degree, and doctor degree. “Living with both parents” and “living only with mother or father, or other situation” was used to evaluate family support. Household income was divided into six groups according to median household incomes in China: None, <4000 RMB/month, 4000–8000 RMB/month, 8001–15,000 RMB/month, 15,001–30,000 RMB/month, and >30,000 RMB/month (1 RMB ≈ 0.16 US dollars).

Children’s daily behavior was an important indicator of physical activity [19] and was associated with cognitive functions [10]. Therefore, it was included as a covariate in the regression models. In this study, the past 2 months of children’s daily behavior were measured using four items from the Chinese Child Behavior Checklist for Preschool Children. The items, completed by their teacher, were as follows: (1) whether the child shows lack of concentration or non-persistent attention; (2) whether the child is introverted and unwilling to talk; (3) whether the child is over-fatigued; and (4) whether the child has slow actions or anenergia. Items were rated by a 3-point scale (0 = not true, 1 = sometimes true, or 2 = often true). Finally, children’s behavior scores were divided into three groups based on the total scores: low (4–6 scores), median (7–9 scores), and high (10–12 scores).

Cardiorespiratory fitness was assessed by the multistage 20 m shuttle run test, which measured cardiorespiratory fitness by running back and forth for 20 m with a starting speed of 8.5 km/h and increasing by 0.5 km/h with each level thereafter (1 min). Maximal performance was determined when the participant failed to follow the pace for two consecutive attempts or stopped due to exhaustion. Results were expressed as laps; one lap corresponded to 20 m. The multistage 20 m shuttle run test is widely used for assessing cardiorespiratory fitness in preschool children and has shown to have a high reliability [20]. Due to the young age of participants, each child had an adult running with them during the process to make sure the test was successfully completed.

### 2.3. Statistical Analyses

Analyses were performed using SPSS version 22.0 (SPSS Inc., Chicago, IL, USA). The normal distribution test was conducted using Kolmogorov–Smirnov test, and variables were described as mean ± SD for the normally distributed variables or median (interquartile ratio, IQR) for non- normally distributed variables. Independent *t* tests, Mann–Whitney *U* tests, or chi-square tests were used to assess sex differences for normally distributed, non-normally distributed, or categorical variables, respectively.

Linear regression analyses were used to explore the association between different intensities of PA and cognitive functions. Variables were transformed to normal distribution using the log or square root method before linear regression, if necessary. To understand total variance explained by different factors, cognitive function regressed in three models: Model 1 LPA and MVPA entered the model and was unadjusted for confounding factors; Model 2 was adjusted for sociodemographic and children’s daily behavior (including age, BMI status, mother’s education, family structure, household income, and child behavior scores); Model 3 was further adjusted for cardiorespiratory fitness. Because PA and physical fitness have been suggested to be strongly linked with sex, our linear regression analyses were stratified for sex. Furthermore, to test the robustness and avoid high correlation between LPA and MVPA causing confusion of our results, LPA, MVPA, and total time engaged in physical activity (TPA, equal to sum of LPA and MVPA) were separately entered into the regression model again following the three steps described above. A two-sided *p* value ≤ 0.05 was considered as statistically significant.

## 3. Results

A total of 325 of 346 participants completed the C-WYCSI test. Among them, 11 preschool children were excluded from analyses because of noncooperation during the test (*n* = 6) or intellectual disability (*n* = 5, FIQ: 54.2–73.8). Characteristics of 260 participants (144 boys; 116 girls) who have complete data of cognitive functions, PA, and confounding factors are shown in Table 1. On average, the age of participants was 57.2 months (55.4% boys), the majority (79.2%) of preschool children were considered as healthy weight according to IOTF, and the percentage of overweight/obesity, LPA, and MVPA in boys were significantly higher than girls. However, the correlation between physical activity and cognitive functions were not significant in girls (*p* > 0.05).

The results showed that LPA, but not MVPA, is significantly correlated with VIQ, PIQ, and FIQ, even when adjusting for several potential confounding factors in the final model in boys. Corresponding standardized coefficients were 0.211, 0.218, and 0.242 (all *p* < 0.05), respectively (Table 2). However, the association between LPA and different categories of IQ were not found in girls in this study. Furthermore, the results were similar with those described above when LPA and MVPA were separately entered into the model; only LPA positively correlated with FIQ, solely in boys (*β =* 0.208, *p* < 0.05). In addition, when TPA replaced LPA and MVPA into the model, the results also suggested TPA positively associated with VIQ, and FIQ, solely in boys (*β =* 0.236 and 0.179, all *p* < 0.05).

## 4. Discussion

In this cross-sectional study, the major finding was that LPA was significantly and positively associated with intelligence quotient in boys, but not in girls. Based on the evidence available, the most recent systematic review concluded that there is a positive association between PA and cognitive function in children, although more studies are needed to identify the effects of different types, amounts, and frequencies of PA on cognitive function [6]. Moreover, in general, MVPA was recommended for children and adolescents because of the “intensity threshold” of PA benefit [21]. However, findings from this study are not in agreement with the results of previous studies, which found only LPA was evidently correlated with cognitive function, as measured by standardized IQ testing, in preschool children. A potential reason has been suggested to explain why different intensities of PA may play a different role among different age groups. One of the hypotheses of PA’s effect on cognitive function was mediated by cardiorespiratory fitness [22], which increased responding to LPA in preschool children but may need higher intensity stimulation in children and adolescents. Nevertheless, it is not suggested that we can ignore the importance of MVPA, although only LPA is shown to be correlated with cognition in this study. Further, total minutes of PA (TPA, sum of LPA and MVPA) also presented a notable association with VIQ and FIQ when TPA took the place of LPA and MVPA in the model. Moreover, the current guidelines of PA recommend accumulating at least 180 min of PA daily at any intensity, and especially highlight the importance of TPA for preschool children [23]. Engaging in both LPA and MVPA lead to increases in the amount of TPA and, therefore, neither of them can be ignored.

The positive association between PA and cognitive functions solely found in boys in this study is also contrary to a recent review article, which showed the sex-dependent effect was more significant in girls [1]. A possible explanation for the findings was the lower level of cardiorespiratory fitness in girls at baseline which could result in more apparent physiological effect in the analysis [24]. Considering the possible influencing factors in this study, there were several explanations for our current results. First, boys engaged in more PA than girls, possibly having a dose–response effect, allowing boys to accrue greater cognitive function benefits. Especially, accumulating evidence from animal to human studies demonstrated that engaging in more physical activity can increase expression and concentration of brain-derived neurotrophic factor in hippocampus [25], which has been to play a crucial role in brain plasticity and functions [26]. Second, contrary to adolescents, preschool children, especially boys, were found to have lower levels of cardiorespiratory fitness in this study. This may have been a physiological effect derived from boys’ PA stimulation. Furthermore, hypothalamic–pituitary–adrenal (HPA) axis response to PA is sex-dependent, possibly causing the sex-dependent effect [27]. For example, higher activation of HPA axis to PA in boys initiates a number of physiological changes, such as stimulating protein synthesis, which serves as the basis for a number of hormones, including adrenocorticotropic hormone (ACTH) and bendorphin that improve cognition, behavior response, and homeostatic challenges [28]. Additionally, sex difference is also a result of genetic variation, parental and familial factors, and one’s acquired behaviors and perceptions, with the latter often shaped by unique experiences at the individual, parental, and familial levels [29].

Our study has several strengths. First, PA was measured using an accelerometer, which avoided the recall bias of proxy report by parents or teachers. Second, several potential confounding factors were adjusted in the statistical analyses. Third, our results were strengthened by combining and separating LPA and MVPA into linear regression models. However, our study also has several limitations. First, for feasibility, we used a convenience sample in this observational study. Second, our cross-sectional study design has limited ability to draw a causal relation of our findings. We cannot illustrate whether PA improves cognitive functions in boys or whether preschool aged boys with high levels of cognitive function simply tend to participant in more PA. Last, the accelerometer was worn over the hip, which limited the ability to capture activities with little displacement of the body, such as cycling. However, the hip was probably the best placement to capture whole-body movements and was also the site most often used by various studies [30].

## 5. Conclusions

In conclusion, our findings suggest PA has a significant and positive association with cognitive functions in boys, especially LPA. On the basis of this study, we indicated the benefit of cognitive function derived from LPA and recommend sex-dependent responses should be considered in future studies. Moreover, more prospective and intervention studies are needed to clarify the causal relation and mechanisms of our observed sex-specific effect.

## Figures and Tables

**Table 1:** Characteristics of the analyzed sample.

	Total (*n* = 260)	Boys (*n* = 144)	Girls (*n* = 116)	*p* for Sex
Age (month)	57.2 ± 5.4	57.6 ± 5.4	56.7 ± 5.3	0.200
BMI (kg/m^2^)	16.2 ± 1.9	16.5 ± 1.9	15.9 ± 1.8	**0.001**
Normal	206	106	100	**0.013**
Overweight/Obesity	54	38	16	
Mother’s education				0.236
Less than high school	10	3	7	
High school	44	28	16	
College/associate degree	82	42	40	
Bachelor’s degree	94	57	37	
Master’s degree	19	8	11	
Doctor degree	11	6	5	
Family structure				0.502
Living with both parents	251	140	111	
Others	9	4	5	
Household income (RMB/month)				0.866
<4000	5	3	2	
4000–8000	42	22	20	
8001–15,000	115	65	50	
15,001–30,000	80	46	34	
>30,000	18	8	10	
Child behavior scores (count)				<0.001
Low (4–6 scores)	165	77	88	
Median (7–9 scores)	88	60	28	
High (10–12 scores)	7	7	0	
Cardiorespiratory Fitness (lap)	11.0 (10–14)	11 (9.25–14.0)	12 (10.0–14.75)	0.328
Physical activity (min/day)				
LPA	98.4 ± 17.1	100.6 ± 17.9	95.6 ± 15.7	**0.021**
MVPA	71.8 ± 17.3	74.1 ± 18.7	69.0 ± 15.0	**0.021**
Cognitive function				
VIQ	23 (19.0–26.0)	22 (19.0–26.0)	23 (19.0–26.0)	0.558
FIQ	25 (22.0–27.0)	25 (21.0–27.0)	25 (23.0–27.75)	0.255
FIQ	110.5 ± 12.4	110.0 ± 13.0	111.3 ± 11.6	0.370

Note: BMI, body mass index; LPA, light physical activity; MVPA, moderate to vigorous physical activity; VIQ, Verbal Intelligence Quotient; PIQ, Performance Intelligence Quotient; FIQ, Full Intelligence Quotient. The mean ± SD or median (interquartile ratio, IQR) was reported for normal or non-normal distribution variables.

**Table 2:** Linear regression analyses between cognitive function and physical activity.

Predictor Variables	VIQ		PIQ		FIQ	
	*β*	95% CI	*β*	95% CI	*β*	95% CI
Boys						
Model 1 *						
LPA	0.203	−0.009, 0.407	0.191	−0.031, 0.416	**0.224**	**0.008, 0.441**
MVPA	0.162	−0.048, 0.356	−0.001	−0.218, 0.217	0.082	−0.130, 0.290
*R*^2^	0.099		0.022		0.069	
Model 2 ^†^						
LPA	**0.197**	**0.009, 0.377**	0.188	−0.019, 0.398	**0.218**	**0.029, 0.408**
MVPA	0.064	−0.120, 0.242	−0.076	−0.280, 0.131	−0.016	−0.202, 0.171
*R*^2^	0.300		0.152		0.291	
Model 3 ^‡^						
LPA	**0.211**	**0.018, 0.395**	**0.218**	**0.007, 0.433**	**0.242**	**0.048, 0.435**
MVPA	0.043	−0.150, 0.232	−0.122	−0.335, 0.096	−0.051	−0.245, 0.146
*R*^2^	0.297		0.157		0.293	
Girls						
Model 1 *						
LPA	−0.028	−0.271, 0.212	0.064	−0.161, 0.289	0.007	−0.225, 0.240
MVPA	−0.009	−0.265, 0.245	0.108	−0.124, 0.353	0.037	−0.206, 0.286
*R*^2^	−0.017		0.006		−0.016	
Model 2 ^†^						
LPA	−0.073	−0.311, 0.156	0.025	−0.205, 0.255	−0.038	−0.268, 0.191
MVPA	−0.025	−0.276, 0.220	0.103	−0.134, 0.353	0.029	−0.212, 0.274
*R*^2^	0.090		0.011		0.060	
Model 3 ^‡^						
LPA	−0.023	−0.252, 0.203	0.027	−0.206, 0.261	−0.003	−0.232, 0.226
MVPA	−0.143	−0.414, 0.092	0.097	−0.157, 0.363	−0.053	−0.312, 0.196
*R*^2^	0.157		0.002		0.087	

Note: *β*, standardized coefficients; *R*^2^, adjusted R square; LPA, light physical activity; MVPA, moderate to vigorous physical activity; VIQ, Verbal Intelligence Quotient; PIQ, Performance Intelligence Quotient; FIQ, Full Intelligence Quotient; the *p* values less than 0.05 are bolded; * Model 1: unadjusted; ^†^ Model 2: adjusted for age, BMI status, mother’s education, family structure, household income, and child behavior scores; ^‡^ Model 3: further adjusted for cardiorespiratory fitness, which was log-transformed before being entered into the model.
